# The Usefulness of the Eosinophilic Esophagitis Histology Scoring System in Predicting Response to Proton Pump Inhibitor Monotherapy in Children with Eosinophilic Esophagitis

**DOI:** 10.3390/diagnostics13223445

**Published:** 2023-11-15

**Authors:** Jovan Jevtić, Nina Ristić, Vedrana Pavlović, Jovana Svorcan, Ivan Milovanovich, Milica Radusinović, Nevena Popovac, Ljubica Simić, Aleksandar Ćirović, Miloš Đuknić, Maja Životić, Nevena Poljašević, Danilo Obradović, Jelena Filipović, Radmila Janković

**Affiliations:** 1 Institute of Pathology, Faculty of Medicine, University of Belgrade, 11000 Belgrade, Serbia; ljubica.simic87@gmail.com (L.S.); djuknicmilos996@gmail.com (M.Đ.); majajoker@gmail.com (M.Ž.); drdaniloobradovic@gmail.com (D.O.); vjesticaj@gmail.com (J.F.); 2 Department of Gastroenterology, Hepatology and GI Endoscopy, University Children’s Hospital, 11000 Belgrade, Serbia; nina.ristic13@gmail.com (N.R.); svorcanjovana@yahoo.com (J.S.); imilovanovich@gmail.com (I.M.); milicaradusinovic@hotmail.com (M.R.); nevena.popovac@gmail.com (N.P.); 3 Institute of Medical Statistics and Informatics, Faculty of Medicine, University of Belgrade, 11000 Belgrade, Serbia; vedrana.pavlovic@med.bg.ac.rs; 4 Institute of Anatomy, Faculty of Medicine, University of Belgrade, 11000 Belgrade, Serbia; aleksandar.cirovic.7@gmail.com; 5 Department of Pathology, University Clinical Center Tuzla, 75000 Tuzla, Bosnia and Herzegovina; nevena.poljasevic@gmail.com

**Keywords:** eosinophilic esophagitis, pediatrics, EoEHSS, score, EREFS

## Abstract

Background: Eosinophilic esophagitis (EoE) is an immune-mediated esophageal disease with rising incidence. While proton pump inhibitors (PPIs) are the first-line treatment, a significant proportion of patients do not respond. This study aimed to determine if the EoE Histology Scoring System (EoEHSS) can predict PPI responsiveness. Methods: A cross-sectional study was conducted on 89 pediatric patients diagnosed with EoE between 2016 and 2022. Patients were categorized into PPI responders (PPIREoE) and non-responders (PPINREoE) based on post-treatment biopsies. EoEHSS values from biopsies of the esophagus (distal, middle, and proximal segments) were compared between the two groups. Results: No significant differences in EoEHSS scores were observed for the distal and proximal esophagus between the groups. However, the middle esophagus showed a significantly higher EoEHSS grade score in the PPINREoE group, indicating a more pronounced disease severity. Specific histological features, particularly eosinophilic abscesses and surface layering of the middle segment of the esophagus, were significantly different between the groups. Conclusions: Performing a biopsy of each esophageal segment, particularly the middle, is crucial for diagnostic precision and predicting PPI responsiveness. The EoEHSS can serve as a valuable tool in predicting therapy response, emphasizing the need for personalized therapeutic approaches in EoE management.

## 1. Introduction

Eosinophilic esophagitis (EoE) is an immune-mediated disease characterized by esophageal dysfunction and a peak eosinophil count (PEC) ≥ 15 eosinophils per high-power field (HPF) on histological examination, in the absence of other conditions and diseases that could cause eosinophilia of the esophagus [1]. The incidence of EoE is consistently increasing, in both children and adults. Although it was initially believed that the incidence was rising due to increased awareness and appropriate diagnostic evaluation, recent studies have shown a real increase in the occurrence of EoE [2,3]. EoE is usually not a life-threatening disease, but nevertheless requires timely treatment. Prolonging the time of diagnosis or applying inappropriate therapy can lead to complications [1] such as esophageal stenosis or perforation, the latter of which can prove fatal [4]. Treatment modalities for EoE include proton pump inhibitors (PPIs), topical corticosteroids, and a strict dietary regimen (the elimination of certain foods). Considering that they are safe, inexpensive, and easily applicable, PPIs are the first–line therapy [5]. However, a certain proportion of patients (51%) with EoE does not respond to PPI treatment and they require a different therapeutic approach [6,7]. Given that there is a population of patients who do not respond to PPIs, predicting the response to these drugs would shorten the time to effective treatment.

In 2017, Collins et al. developed the EoE Histology Scoring System (EoEHSS) which has proven to be superior to peak eosinophil count in terms of diagnosis and patient monitoring [8]. This raises the question whether EoEHSS can also be used to predict response to therapy. The objective of our study is to compare EoEHSS values from biopsies of the distal, middle, and proximal segments of the esophagus, as well as clinical parameters, between two patient groups based on their response to PPI therapy, with the aim of predicting therapy response.

## 2. Materials and Methods

### 2.1. Study Design and Patient Selection

This cross-sectional study included 89 pediatric patients, aged between 0 and 18 years, who were diagnosed and treated for EoE at a tertiary healthcare center between 2016 and 2022. The diagnosis of EoE was made according to guidelines [1]. Biopsies were taken from all segments of the esophagus (proximal, middle, distal). A minimum of four tissue samples were taken from at least two locations, typically proximally and distally. In cases where all three segments of the esophagus were biopsied, a minimum of six tissue samples were taken. This sampling approach was applied both during the initial diagnosis and in the evaluation of the PPI therapy’s effectiveness. After the diagnosis was established, all patients received PPI treatment at a dosage of 1–2 mg/kg per day. Three months after treatment, a follow-up endoscopy with multiple biopsies was performed to evaluate the treatment’s effectiveness. Based on their response to the PPI therapy, patients were divided into two groups: responders (PPIREoE) and non-responders (PPINREoE). Responders were defined as those patients who had fewer than 15 eosinophils per HPF within their esophageal biopsies. Analyses for this study were conducted based on data from the initial endoscopy and initial biopsies. We analyzed 151 tissue samples (35 samples of PPIREoE and 116 samples of PPINREoE) from the distal esophageal segment, 64 tissue samples (28 samples of PPIREoE and 36 samples of PPINREoE) from the middle esophageal segment, and 148 samples (32 samples of PPIREoE and 116 samples of PPINREoE) from the proximal esophageal segment. The inclusion criteria for patients into the study were: at least two esophageal biopsies, before and after the administration of therapy. The exclusion criteria were if the patient initially received some other form of therapy other than PPI, if the patient did not undergo a follow up endoscopy with biopsy after treatment, or if the slides were inadequate for analysis because they had faded or for other reasons. This study was conducted with the approval of the Ethics Committee of the Faculty of Medicine, University of Belgrade.

### 2.2. Demographics, Clinical Characteristics and Endoscopy

The data collected included demographic information, allergy details, laboratory values, comorbidities, and symptoms at the time of the initial endoscopy. Endoscopic data related to EoE were collected using the EREFS score, which is actually an acronym derived from the initial letters of the endoscopic features of EoE (Edema, Rings, Exudates, Furrows, and Strictures) [9].

### 2.3. Tissue Processing and Preparation of H&E Slides for Scoring

After performing esophageal endoscopic biopsies, the samples were fixed for 24 h in 10% buffered formalin, then rinsed with distilled water, and subsequently dehydrated in increasing concentrations of alcohol (from 70% to pure alcohol). After dehydration in alcohol, the samples were lipophilized in xylene and, following lipophilization, embedded in paraffin blocks. The obtained paraffin blocks were cut with a standard microtome into sections 3–5 μm thick. The sections were further stained with hematoxylin and eosin (H&E). 

### 2.4. Biopsy Scoring

**Figure 1:**
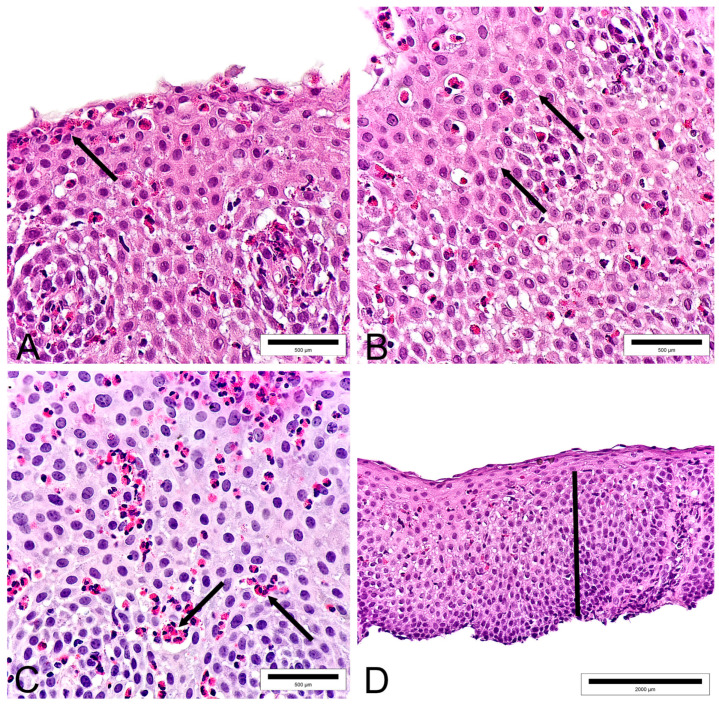
This figure represents a part of the scoring methodology, displaying histological characteristics of EoE that have been scored. ((**A**)—Eosinophil surface layering (arrow), magnification ×100; (**B**)—Dilated intercellular spaces (arrow), magnification ×100; (**C**)—Eosinophilic abscesses (arrow), magnification ×100; (**D**)—Basal zone hyperplasia (The line shows the portion of the esophageal epithelium affected by BZH), magnification ×40; On each image, more than 15 eosinophils per high power field (Eo/HPF) are present).

Biopsy scoring was carried out according to the modified validated EoEHSS system developed by Collins et al. [8]. The EoEHSS encompasses more than just eosinophil count; it also considers various histological characteristics of EoE. This scoring system includes eight characteristics: peak eosinophil count (PEC), basal zone hyperplasia (BZH), eosinophilic abscesses (EA), eosinophil surface layering (SL), dilated intercellular spaces (DIS), surface epithelial alterations (SEA), lamina propria fibrosis (LPF), and dyskeratotic epithelial cells (DEC). Due to their presence in a limited number of biopsies, LPF, SEA, and DEC were excluded. Therefore, the scoring was based on PEC, EA, SL, BZH, and DIS (Figure 1). A grade and stage were assigned to each of the aforementioned characteristics [8]. For each histological feature, grade and stage values were determined semi-quantitatively, ranging from 0 to 3 (Table 1 and Table 2). If the maximum values for grade and stage for each biopsy feature of a patient were 3, then the maximum possible score for grade and stage would be 15. The final score would be calculated by dividing the given biopsy’s grade and stage score by the maximum possible values. Scoring was performed using an Olympus BX43 microscope (Pittsford, NY, USA).

### 2.5. Statistical Analysis

To characterize the study sample, we used descriptive statistics. For numerical variables, we calculated means, medians, standard deviations, and percentiles. For categorical variables, we determined the numbers and their respective percentages. The Pearson chi-squared test or Fisher’s exact test were used to evaluate associations between categorical data. The Student’s *t*-test or the Mann-Whitney U test were used for numerical data to evaluate differences between responders and non-responders. Univariate logistic regression analysis was used to establish factors related to overall therapy response. In all analyses, the level of statistical significance was set at *p* ≤ 0.05. SPSS version 25 statistical software (Chicago, IL, USA) was used to perform the statistical analysis. 

## 3. Results

### 3.1. Demographic and Clinical Characteristics

A total of 89 pediatric patients with EoE were included in the study. The average age of study participants was 12.1 ± 3.8 years and more than half were male (78.7%). The youngest patient included was 2 and the eldest was 18 years old. There was no significant age or gender differences between the PPIREoE and PPINREoE groups. Additionally, no differences were observed in terms of allergic factors (Table 3). Regarding symptoms, pain and dyspeptic complaints were more prevalent among PPIREoE, while regurgitation and food impaction were more common among PPINREoE. Vomiting and dysphagia were almost equally represented in both groups. Interestingly, comorbidities were exclusively observed in the non-responder group of patients.

### 3.2. Histological Characteristics

The final EoEHSS grade score for distal segment biopsies was 0.6 (0.5–0.7) for the PPINREoE group and 0.5 (0.4–0.7) for the PPIREoE group, indicating a slight but not statistically significant difference in the overall disease severity score between the two groups. A similar result was observed in biopsies from the proximal segment where the score was 0.6 (0.3–0.7) for the PPINREoE group and 0.4 (0.2–0.6) for the PPIREoE group. When we compared individual components of EoEHSS for grade, no statistically significant difference was observed between PPINREoE and PPIREoE in both distal and proximal segments of the esophagus. 

**Figure 2:**
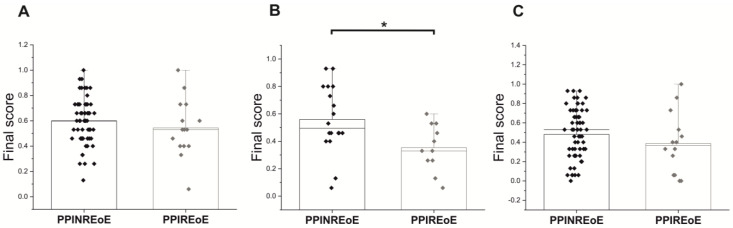
EoEHSS final score for grade of PPINREoE and PPIREoE; (**A**) EoEHSS final score of the distal esophagus; (**B**) EoEHSS final score of the middle esophagus; (**C**) EoEHSS final score of the proximal esophagus. * *p* < 0.05.

Interestingly, the final EoEHSS grade score for the middle segment was 0.5 (0.5–0.8) for the PPINREoE group, which was significantly higher than the 0.3 (0.3–0.5) observed in the PPIREoE group (*p* = 0.037), indicating a more pronounced severity in the PPINREoE group. Similar results were obtained when comparing individual components of the EoEHSS with respect to grade, revealing a more pronounced disease severity in the PPINREoE group. Specifically, EA and SL demonstrated a statistically significant difference compared with the PPIREoE group (*p* = 0.044 and *p* = 0.046, respectively) (Table 4) (Figure 2).

**Figure 3:**
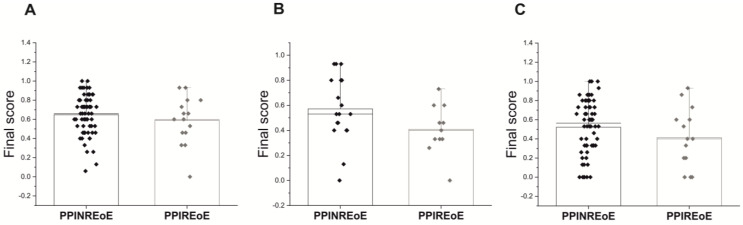
EoEHSS final score for stage of PPINREoE and PPIREoE; (**A**) EoEHSS final score of the distal esophagus; (**B**) EoEHSS final score of the middle esophagus; (**C**) EoEHSS final score of the proximal esophagus.

None of the EoEHSS stage scores showed a statistically significant difference between PPINREoE and PPIREoE for the proximal, distal, or middle segments of the esophagus. Analysis of the individual EoEHSS parameters for disease stage yielded results consistent with those observed for disease grade. Notably, significant differences between PPINREoE and PPIREoE were identified in the EA values from biopsies of the middle segment and BZH values from the proximal segment, suggesting a more extensive disease distribution in patients unresponsive to therapy (Table 5) (Figure 3).

## 4. Discussion

The consistent increase in the incidence of EoE necessitates research regarding its etiology, pathogenesis, as well as the refinement of diagnostics and therapy. The primary objective of EoE treatment is not only to alleviate disease symptoms and enhance patients’ quality of life but also to prevent potential complications, including those that could be life-threatening. Delay in treatment or application of inadequate therapy carries the risk of disease complications. The aim of this study is to predict the response to PPI therapy as the first-line treatment in patients with EoE, with the goal of initially selecting the therapy to which the patient will best respond. Additionally, there is still no method to predict the response to PPI therapy, which would enable selection of the appropriate treatment at the outset. Our study, encompassing 89 pediatric patients, provides valuable insights into the clinical and histological characteristics of EoE in relation to PPI responsiveness.

Taking into account the response to PPI therapy, our study showed a lower rate of response compared with most studies. However, there are also studies in the pediatric population that have shown a similar response rate [10]. Such findings can be explained by poorer compliance with therapy, considering that a significant number of adolescents were included in the study, where compliance with therapy is generally lower. Additionally, it has been demonstrated that the response to PPI therapy in the pediatric population is generally poorer compared with adults [11]. 

From a demographic perspective, our cohort predominantly comprised males, consistent with previous literature that has identified a male preponderance in EoE [12]. The age range of our study participants, spanning from 2 to 18 years, highlights the broad spectrum of pediatric ages at which EoE can manifest. Notably, the absence of significant age or gender differences between the PPIREoE and PPINREoE groups suggests that these demographic factors might not play a pivotal role in determining PPI responsiveness.

The clinical presentations of EoE are known to be diverse, and our study confirms this heterogeneity. While pain and dyspeptic complaints were more common in the PPIREoE group, regurgitation and food impaction were predominant in the PPINREoE group. This divergence in symptomatology could be attributed to variations in esophageal mucosal involvement, eosinophilic infiltration, or other inflammatory processes, which might be influenced by genetic, environmental, or immunological factors. The equal representation of vomiting and dysphagia in both groups, however, suggests that these symptoms might not be reliable indicators of PPI responsiveness. The exclusive presence of comorbidities in the non-responder group is intriguing and raises questions about potential associations between comorbid conditions and PPI resistance. Given that the comorbidities in our study group were highly diverse (asthma, epilepsy, Hashimoto’s thyroiditis, kidney agenesis, cerebellar medulloblastoma, etc.), even after an extensive literature search, we were unable to find a connection with a lack of response to therapy. Therefore, further research on this topic is essential.

Although nearly fifty years have passed since EoE was first described, there are still no clear protocols regarding the number and localization of biopsies necessary for diagnosis [13,14]. While various studies emphasize the importance of performing a biopsy on both the proximal and distal segments of the esophagus for diagnosis [15], others highlight the significance of also sampling the middle segment of the esophagus [16,17]. The only point on which most researchers agree on is taking a larger number of biopsies from different locations (polytopic biopsies). Our study underscores the critical need for a standardized approach to biopsy protocols in EoE diagnosis. The ambiguity surrounding the optimal number and location of biopsies highlights a significant gap in the current understanding and approach to EoE diagnosis and management. The scores (grade and stage) for the distal and proximal segment did not show significant differences between the two groups. Even though they cannot be used to predict the response to PPI, biopsies of the distal and proximal esophageal segments are essential for the diagnosis of EoE and for differentiation from GERD. However, the middle segment of the esophagus emerged as a potential region of interest. In a recent study by Hiermath et al., it was shown that most histological features of EoE, except for DIS, are not uniformly represented in all parts of the esophagus, regardless of whether the EoE is active or in remission [18]. Lin et al. demonstrated that EoEHSS scores can significantly vary between the distal and middle segments of the esophagus in both the population of patients with active EoE and in patients in remission. Additionally, it has been shown that EoEHSS scores were not always higher in the distal segment of the esophagus, suggesting that such distribution might be associated with different types of EoE, varying symptomatology, and distinct therapeutic requirements and responses [17]. The significantly higher EoEHSS grade score for the PPINREoE group in the middle segment in our study suggests that this region might be more susceptible to severe eosinophilic infiltration, especially in non-responders to PPI therapy. The pronounced differences in individual components, particularly EA and SL, further emphasize the importance of performing a biopsy on the middle segment of the esophagus. Additionally, Lin et al. have shown that different segments of the esophagus do not undergo simultaneous repair, with the mid-esophagus, where changes related to damage have been observed to persist for a longer period [17]. Interestingly, despite the differences observed in the grade scores, the EoEHSS stage scores did not offer a clear distinction between the two groups across all esophageal segments. By analyzing individual score characteristics with respect to stage, a statistically significant difference between PPINREoE and PPIREoE was observed in the EA values of the middle segment and BZH values of the proximal segment, potentially indicating that the distribution of the disease may be significant in terms of response to therapy.

While this study brings new insights, it also has its limitations, primarily in terms of its retrospective nature, a small sample size and univariate analysis. Future studies should aim to validate our findings in larger cohorts and explore the underlying mechanisms driving the observed differences between PPI responders and non-responders.

Further research could employ immunohistochemistry to assess eosinophil activity, potentially highlighting differences between these groups. Molecular investigations are also essential for a more in-depth understanding. The role of genetic factors in determining the response to PPI therapy also cannot be overlooked. Genetic polymorphisms might influence the pharmacodynamics and pharmacokinetics of PPIs, thereby affecting the therapeutic outcomes. Understanding the genetic makeup of the patients could potentially aid in personalizing the treatment strategies, ensuring more effective and efficient management of EoE. Additionally, examining the intercellular connections in these two patient groups is crucial. The epithelium’s permeability itself may be linked to the response to therapy, necessitating a comprehensive exploration in this aspect. Research on the esophageal microbiota could significantly contribute to understanding the pathogenesis and selection of therapy for EoE. It has been shown that the esophageal microbiota in EoE patients is altered, with an increase in Haemophilus and a decrease in Firmicutes [19,20,21]. Additionally, a study conducted by Parashette et al. demonstrated significant differences in microbiota among EoE patients in terms of their response to PPI therapy, particularly in the context of the *Bacteroidetes* phylum [22]. Therefore, further investigations of the microbiome may hold significance in predicting the response to PPI therapy.

## 5. Conclusions

The EoEHSS score was successfully applied for the first time in order to predict the response to PPI therapy in children with EoE. EoEHSS grade score of the middle esophageal segment in PPINREoE was significantly higher compared with PPIREoE. Although some authors recommend performing a biopsy of two regions of the esophagus (most commonly the proximal and distal segments), our study points to the need for all segments of the esophagus (proximal, middle and distal) to be examined, particularly the middle. This increases diagnostic precision and facilitates therapeutic personalization through prediction of PPI responsiveness. Additionally, besides assessing the response to various forms of therapy, EoEHSS can also be an excellent tool in predicting the response.

## Figures and Tables

**Table 1:** Grading within the EoEHSS.

Grade Score	
Peak eosinophil count (PEC)	
0	PEC 0
1	PEC < 15/HPF
2	PEC 15–59/HPF
3	PEC > 60/HPF
Basal zone hyperplasia (BZH)	
0	BZH not present
1	BZH occupies >15% but <33% of the total thickness
2	BZH occupies 33–66% of the total thickness
3	BZH occupies >66% of the total thickness
Eosinophilic abscesses (EA)	
0	EA not present
1	EA consists of 4–9 eosinophils
2	EA consists of 10–20 eosinophils
3	EA consists of >20 eosinophils
Eosinophil surface layering (SL)	
0	SL not present
1	SL consists of 3–4 eosinophils
2	SL consists of 5–10 eosinophils
3	SL consists of >10 eosinophils
Dilated intercellular spaces (DIS)	
0	DIS not observed at any magnification
1	DIS are observed only at 400× magnification
2	DIS are observed at 200× magnification
3	DIS are observed at 100× magnification or lower

**Table 2:** Staging within the EoEHSS.

Stadium Score	
Peak eosinophil count (PEC)	
0	PEC 0–14/HPF
1	PEC ≥ 15/HPF in <33% HPFs
2	PEC ≥ 15/HPF in 33–66% HPFs
3	PEC ≥ 15/HPF in >66% HPFs
Basal zone hyperplasia (BZH)	
0	BZH not present
1	BZH of any grade > 0 occupying < 33% of the biopsy epithelium
2	BZH of any grade > 0 occupying 33–66% of the biopsy epithelium
3	BZH of any grade > 0 occupying > 66% of the biopsy epithelium
Eosinophilic abscesses (EA)	
0	EA not present
1	EA of any grade > 0 occupying < 33% of the biopsy epithelium
2	EA of any grade > 0 occupying 33–66% of the biopsy epithelium
3	EA of any grade > 0 occupying > 66% of the biopsy epithelium
Eosinophil surface layering (SL)	
0	SL not present
1	SL of any grade > 0 occupying < 33% of the biopsy epithelium
2	SL of any grade > 0 occupying 33–66% of the biopsy epithelium
3	SL of any grade > 0 occupying > 66% of the biopsy epithelium
Dilated intercellular spaces (DIS)	
0	DIS not present
1	DIS of any grade > 0 occupying < 33% of the biopsy epithelium
2	DIS of any grade > 0 occupying 33–66% of the biopsy epithelium
3	DIS of any grade > 0 occupying > 66% of the biopsy epithelium

**Table 3:** Demographic and clinical characteristics of PPINREoE and PPIREoE.

	Total (*n* = 89)	Response to Therapy		
		PPINREoE (*n* = 72)	PPIREoE (*n* = 17)	*p*
Gender, *n* (%)				0.284
Male	70 (78.7)	55 (76.4)	15 (88.2)	
Female	19 (21.3)	17 (23.6)	2 (11.8)	
Age, mean ± sd	12.1 ± 3.8	12.3 ± 3.8	11.7 ± 3.5	0.594
Atopy, *n* (%)	20 (22.5)	17 (23.6)	3 (17.6)	0.596
Food Alergy—SPT, *n* (%)	12 (13.5)	10 (13.9)	2 (11.8)	0.818
Food Alergy—IGE, *n* (%)	24 (27.0)	20 (27.8)	4 (23.5)	0.723
Inhalation allergy panel test, *n* (%)	23 (25.8)	19 (26.4)	4 (23.5)	0.809
Comorbidities, *n* (%)	17 (19.1)	17 (23.6) *	0 (0.0)	0.026 *
Regurgitation, *n* (%)	4 (4.5)	4 (5.6)	0 (0)	0.320
Dysphagia, *n* (%)	31 (34.8)	25 (34.7)	6 (35.3)	0.964
Impaction, *n* (%)	34 (38.2)	29 (40.3)	5 (29.4)	0.407
Pain, *n* (%)	21 (23.6)	14 (19.4)	7 (41.2)	0.058
Dyspepsia, *n* (%)	13 (14.6)	8 (11.1)	5 (29.4)	0.055
Vomiting, *n* (%)	12 (13.5)	10 (13.9)	2 (11.8)	0.818
EREFS, median (25th–75th percentile)	2 (1–2)	2 (1–2)	2 (0.5–2)	0.291

* Statistically significant. PPINREoE: proton pump inhibitor non-responders; PPIREoE: proton pump inhibitor responders; SPT: skin prick test; EREFS: endoscopic reference score.

**Table 4:** EoEHSS final score and individual components for grade of PPINREoE and PPIREoE.

EoEHSS	Response to Therapy		OR	*p*
	PPINREoE	PPIREoE		
DG (*n* = 78)				
PEC	2 (2–3)	2 (2–3)	0.676	0.403
BZH	2 (2–3)	2 (2–3)	0.694	0.296
EA	1 (0–1)	0 (0–1)	0.882	0.734
SL	1 (0–2)	1 (0–1)	0.814	0.495
DIS	3 (2–3)	3 (2–3)	0.755	0.428
Final score	0.6 (0.5–0.7)	0.5 (0.4–0.7)	0.225	0.327
MG (*n* = 29)				
PEC	3 (2–3)	2 (1–2)	0.392	0.112
BZH	3 (2–3)	1 (1–3)	0.562	0.144
EA	1 (0–1) *	0 (0–0)	0.106	0.044 *
SL	1 (0–2) *	0 (0–0)	0.248	0.046 *
DIS	3 (2–3)	2 (1–3)	0.712	0.305
Final score	0.5 (0.5–0.8) *	0.3 (0.3–0.5)	0.011	0.037 *
PG (*n* = 76)				
PEC	2 (1–3)	2 (0–3)	0.648	0.154
BZH	3 (2–3)	3 (0–3)	0.669	0.147
EA	0 (0–1)	0 (0–1)	0.852	0.683
SL	0 (0–2)	0 (0–1)	0.914	0.763
DIS	3 (1–3)	3 (0–3)	0.759	0.293
Final score	0.6 (0.3–0.7)	0.4 (0.2–0.6)	0.278	0.253

Data are presented as median (25th–75th percentile). * Statistically significant. EoEHSS: eosinophilic esophagitis histology scoring system; PPINREoE: proton pump inhibitor non-responders; PPIREoE: proton pump inhibitor responders; DG: distal esophagus grade; MG: middle esophagus grade; PG: proximal esophagus grade; PEC: peak eosinophil count; BZH: basal zone hyperplasia; EA: eosinophilic abscesses; SL: eosinophil surface layering; DIS: dilated intercellular spaces.

**Table 5:** EoEHSS final score and individual components for stage of PPINREoE and PPIREoE.

EoEHSS	Response to Therapy		OR	*p*
	PPINREoE	PPIREoE		
DS (*n* = 78)				
PEC	3 (2–3)	3 (1–3)	0.831	0.512
BZH	3 (3–3)	3 (3–3)	0.730	0.405
EA	1 (0–2)	0 (0–2)	0.890	0.705
SL	1 (0–2)	1 (0–1)	0.713	0.268
DIS	3 (2–3)	3 (2–3)	0.791	0.546
Final score	0.7 (0.5–0.8)	0.6 (0.5–0.8)	0.290	0.339
MS (*n* = 29)				
PEC	3 (1–3)	1 (0–3)	0.750	0.392
BZH	3 (3–3)	3 (2–3)	0.802	0.570
EA	1 (0–2) *	0 (0–0)	0.126	0.049 *
SL	1 (0–2)	0 (0–0)	0.367	0.077
DIS	3 (2–3)	2 (1–3)	0.784	0.470
Final score	0.5 (0.4–0.8)	0.4 (0.3–0.6)	0.053	0.102
PS (*n* = 76)				
PEC	2 (1–3)	2 (0–3)	0.738	0.206
BZH	3 (2–3)	3 (0–3)	0.623	0.041 *
EA	0 (0–1)	0 (0–1)	0.769	0.469
SL	0 (0–2)	0 (0–1)	0.852	0.614
DIS	3 (1–3)	3 (0–3)	0.923	0.745
Final score	0.6 (0.3–0.7)	0.4 (0.2–0.6)	0.276	0.212

Data are presented as median (25th–75th percentile). * Statistically significant. EoEHSS: eosinophilic esophagitis histology scoring system; PPINREoE: proton pump inhibitor non-responders; PPIREoE: proton pump inhibitor responders; DS: distal esophagus stadium; MS: middle esophagus stadium; PS: proximal esophagus stadium; PEC: peak eosinophil count; BZH: basal zone hyperplasia; EA: eosinophilic abscesses; SL: eosinophil surface layering; DIS: dilated intercellular spaces.

## Data Availability

The data presented in this study are available in the article.
